# Supplementary material for: Predicting visual function by interpreting a neuronal wiring diagram
Source: Nature. 2024 Oct 2;634(8032):113–23. doi: 10.1038/s41586-024-07953-5 (PMC11446822; doi:10.1038/s41586-024-07953-5)
Supplement: Supplementary file 6 — CRF and ERF predictions for individual TmY4 and TmY9 cells. Analogous to Supplementary Data 3, but for TmY target types. Shown are the top four monosynaptic pathways, the strongest pathway passing through each of the top ten intermediary types (ranking from Extended Data Fig. 7), and the trisynaptic pathway Tm1–TmY–Dm3–TmY (see the section entitled Prediction of spatial normalization). [file 41586_2024_7953_MOESM6_ESM.zip › DataS4/TmY9q/720575940632760780.pdf]

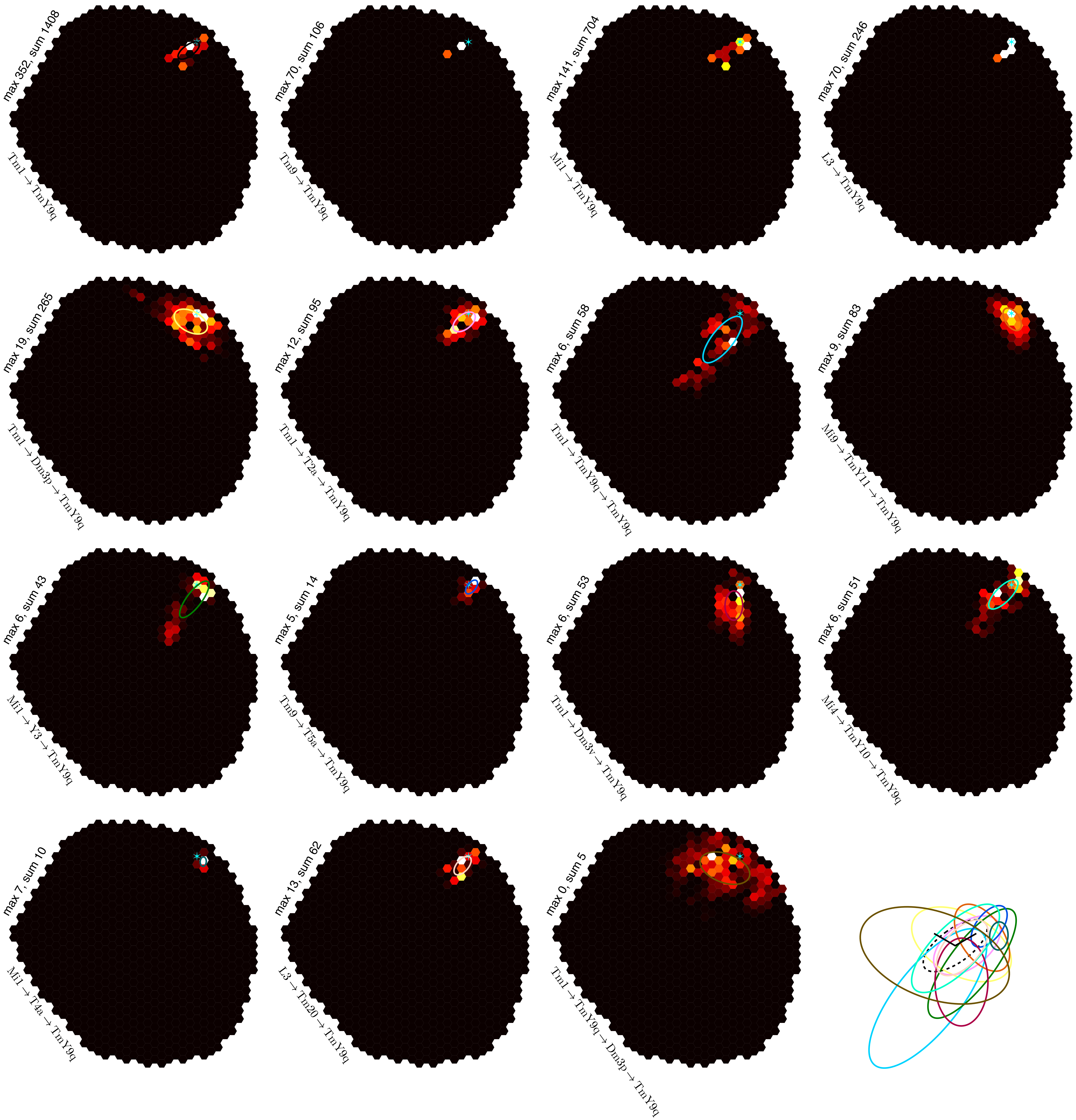
max 352, sum 1408
Tm1 → TmY9q
max 70, sum 106
Tm9 → TmY9q
max 141, sum 704
Mi1 → TmY9q
max 70, sum 246
L3 → TmY9q
max 19, sum 265
Tm1 → Dm3p → TmY9q
max 12, sum 95
Tm1 → T2a → TmY9q
max 6, sum 58
Tm1 → TmY9q → TmY9q
max 9, sum 83
Mi9 → TmY11 → TmY9q
max 6, sum 43
Mi1 → Y3 → TmY9q
max 5, sum 14
Tm9 → T5a → TmY9q
max 6, sum 53
Tm1 → Dm3v → TmY9q
max 6, sum 51
Mi4 → TmY10 → TmY9q
max 7, sum 10
Mi1 → T4a → TmY9q
max 13, sum 62
L3 → Tm20 → TmY9q
max 0, sum 5
Tm1 → TmY9q → Dm3p → TmY9q
